# Etiopathogenesis of Recurrent Aphthous Stomatitis and the Role of Immunologic Aspects: Literature Review

**DOI:** 10.1007/s00005-013-0261-y

**Published:** 2013-11-12

**Authors:** Zuzanna Ślebioda, Elżbieta Szponar, Anna Kowalska

**Affiliations:** 1 Department of Oral Mucosa Diseases, University of Medical Sciences, Bukowska 70, 60-812 Poznan, Poland; 2 Institute of Human Genetics in Poznan, Polish Academy of Sciences, Poznan, Poland; 3 Division Biology of Civilization Diseases, Department of Chemistry and Clinical Biochemistry, University of Medical Sciences, Poznan, Poland

**Keywords:** Recurrent aphthous stomatitis, Etiopathogenesis, Immunologic factors, Cytokines

## Abstract

Recurrent aphthous stomatitis (RAS; recurrent aphthous ulcers; canker sores) belongs to the group of chronic, inflammatory, ulcerative diseases of the oral mucosa. Up to now, the etiopathogenesis of this condition remains unclear; it is, however, considered to be multifactorial. The results of currently performed studies indicate that genetically mediated disturbances of the innate and acquired immunity play an important role in the disease development. Factors that modify the immunologic response in RAS include: food allergies, vitamin and microelement deficiencies, hormonal and gastrointestinal disorders (e.g., celiac disease, Crohn’s disease, ulcerative colitis), some viral and bacterial infections, mechanical injuries and stress. In this paper, we presented the main etiopathogenetic factors of RAS with a special emphasis on the mechanisms of the immune response modification. Moreover, we discussed the crucial clinical symptoms and types of RAS together with epidemiologic data based on the current medical literature reports and our own observations.

## Introduction

Recurrent aphthous stomatitis (RAS; recurrent aphthous ulcers; canker sores) belongs to the group of chronic, inflammatory diseases of the oral mucosa (Field and Allan 2003; McCullough et al. 2007; Rogers 1997; Scully and Porter 2008). The most characteristic symptom of the disease is the recurrent onset of single or multiple painful erosions and ulcers that appear mainly on unattached oral mucosa of the lips, cheeks and tongue. Occasionally the lesions may also be observed on strongly keratinized palatal and gingival mucosa. The eruptions are surrounded by a characteristic erythematous halo and covered with fibrous coating (Chavan et al. 2012; Rogers 1997). Considering the clinical features, three main types of recurrent aphthae can be distinguished: minor aphthae (Mikulicz’s aphthae; MiRAS), major aphthae (Sutton’s aphthae; MaRAS) and herpetiform aphthae (HeRAS) (Burruano and Tortorici 2000; Chattopadhyay and Chatterjee 2007; Natah et al. 2004; Tappuni et al. 2013; Woo and Sonis 1996). The comparison of the most crucial clinical features of RAS according to their classification is presented in Table 1.

**Table 1:** Clinical characteristic of RAS according to their classification

Type of RAS	Clinical feature							
	Size (ø mm)	Type and number of lesions	Depth	Scar	Duration (days)	Peek onset (age)	Frequency compared to other RAS types (%)	Localization
MiRAS	5–10	<10	Shallow	No	10–14	2 life decade	75–90	Non-keratinized oral mucosa. Often: lips, buccal regions, tongue margins
MaRAS	>10	1–3	Deep	Yes	>14	1 and 2 life decade	10–15	Keratinized and non-keratinized oral mucosa. Often: soft palate
HeRAS	<5	>10	Shallow	No	10–14	3 life decade	5–10	Non-keratinized oral mucosa. Often: floor of the mouth, ventral surface of the tongue

Recurrent aphthae appearing simultaneously on the oral and genital mucosa are one of the symptoms of Behçet’s syndrome, a systemic, inflammatory disease with a suspected autoimmune background (Freysdottir et al. 1999; Lehner 1978; Williams and Lehner 1977).

The prevalence of RAS in the general population ranges between 5 and 25 %. Such significant differences have been reported depending on the origin of the examined groups and populations as well as on the studies’ design and methodology (Liang and Neoh 2012; Rogers 1997; Scully et al. 2003; Shashy and Ridley 2000). The presence of aphthae directly during the clinical examination is detected in a lower percentage of the examined subjects when compared with the studies based on the information from the patient’s history. However, the recurrent nature of this condition makes the data from the medical history very relevant in defining the final prevalence of the disease. The comparison of RAS prevalence evaluated based on the results of clinical examination and the information from patient’s history in some international research in correlation with age is presented in Table 2.

**Table 2:** RAS prevalence in the clinical examination and based on the patient’s history in the literature reports

RAS prevalence (%)		Population studies			References
Clinical examination	Patient’s history	Country	Number of participants	Age of participants	
1.2		USA	7,785	17–39	Rivera-Hidalgo et al. (2004)
0.59			9,450	≥40	
1.03		USA	33,994	<1 to ≥60	Chattopadhyay and Chatterjee (2007)
1.2		Turkey	765	5–95	Mumcu et al. (2005)
1.4	18.3	Germany	655	35–44	Reichart (2000)
1.0	6.9		1,367	65–74	
	9.7	Slovenia	555	15–65	Kovač-Kovačič and Skalerič (2000)
	78.1	Jordan	684	13–68	Safadi (2009)
2	27.3	Poland	814	14–18	Górska (1997a)
7.6		Poland	1,216	10–79	Szponar et al. (2006)
10.0		Poland	1,281	ND^a^	Konopka and Mendak (2004)
3.6		Poland	1,500	ND^a^	Bizoń-Wróblewska et al. (1997)

^a^Not defined in the study project

The second life decade is considered as a peak period of the RAS occurrence with the first episode in childhood or in later life stages. As in the other diseases with possible autoimmune-mediated diseases, early age at onset does not necessarily need to be related with a worse prognosis (Amador-Pattaroyo et al. 2012). Based on epidemiologic observations, MaRAS appears more frequently in younger patients, while HeRAS affects most commonly people in third life decade (Chattopadhyay and Chatterjee 2007; Natah et al. 2004; Tappuni et al. 2013; Woo and Sonis 1996). The mechanism of the development of particular type of RAS in correlation with patient’s age has not been clearly understood so far. Although the RAS is generally a common oral mucosal condition; herpetiform type is observed rarely, which makes the comparisons between the types’ occurrence difficult, as most of the reports refer to MaRAS or MiRAS. More profound analysis in this matter is definitely indicated. Generally, the severity and frequency of the episodes vary individually; however, it usually decreases with age, as presented in Table 2 (McCullough et al. 2007; Reichart 2000; Shulman 2004; Sook-Bin and Sonis 1996; Wolach et al. 2012). The reduced occurrence of RAS in elderly in comparison to young patients may partially result from age-dependent alterations in the innate and the adaptive components of the immune system, described as immunosenescence and “inflamm-aging” (DeVeale et al. 2004; Franceschi et al. 2000; Plowden et al. 2004; Senovilla et al. 2013). In elderly, the neutrophils’ chemotactic and phagocytic capacity decreases and the reduced proportion of naive T cells relative to their memory counterparts occurs (Maheaswari et al. 2013; Stankiewicz and Stasiak-Barmuta 2011). Apart from the shifts in population types, immune cells also exhibit altered cytokine production and responsiveness, reduced proliferative responses, signal transduction defects and diminished antigen recognition (Campisi et al. 2009; Maheaswari et al. 2013; Wardzyńska and Kowalski 2009). The prevalence of autoimmune diseases is relatively low in elderly patients also due to age-related increase in peripheral CD4^+^CD25^high^FOXP3^+^ T-regulatory cells (Vadasz et al. 2013). This process may also be involved in RAS.

Apart from the age-related differences in the prevalence, the condition is found three times more often in white citizens than in Afro-Americans (Matranga et al. 2012). Also non-smoking subjects are more prone to develop RAS in comparison to tobacco smokers and smokeless tobacco users (Chattopadhyay and Chatterjee 2007; Grady et al. 1992; Rivera-Hidalgo et al. 2004). Many epidemiologic studies confirmed the higher incidence of RAS in people with a high socio-economic status (McCullough et al. 2007; Rivera-Hidalgo et al. 2004). Also females seem to be in a higher risk of the disease development in comparison to males (McCullough et al. 2007; Rivera-Hidalgo et al. 2004).

**Fig. 1:**
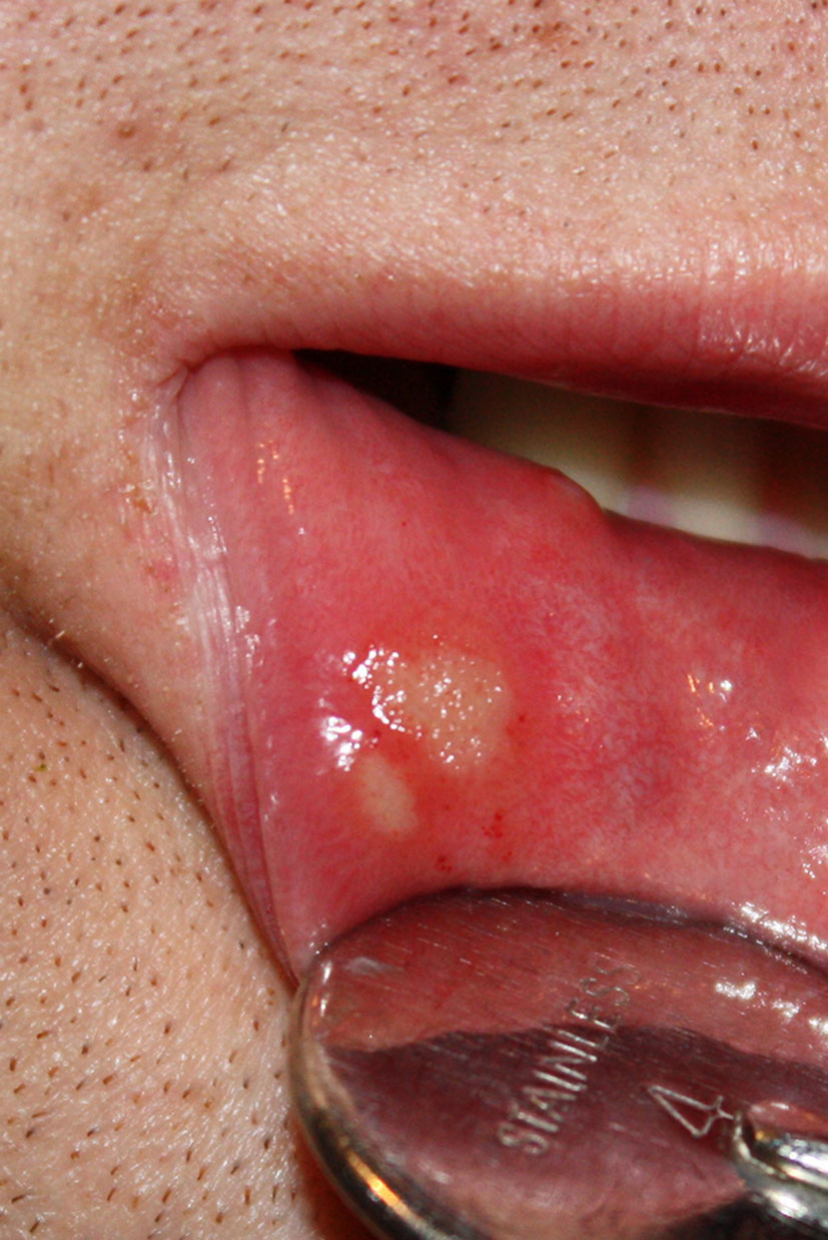
Minor (Mikulicz) aphtha on the lower lip in a patient of the Department of Oral Mucosa Diseases

Minor (Mikulicz) aphtha on the lower lip in a male patient of the Department of Oral Mucosa Diseases is presented in Fig. 1.

## Etiopathogenesis of RAS

The etiopathogenesis of RAS so far remains not fully understood. The potential trigger factors include: genetic predisposition, viral and bacterial infections, food allergies, vitamin and microelement deficiencies, systemic diseases (e.g., celiac disease, Crohn’s disease, ulcerative colitis, AIDS), increased oxidative stress, hormonal defects, mechanical injuries and anxiety (Bilgili et al. 2013; Field and Allan 2003; Koybasi et al. 2006; McCullough et al. 2007; Natah et al. 2004; Scully and Porter 2008). Recently, also the atopic background of the condition has been suggested (Veller-Fomasa and Gallina 2006). In genetically predisposed patients, the effect of certain trigger factors initiates the cascade of proinflammatory cytokines, directed against selected regions of the oral mucosa. The microscopic observation of the aphtha region reveals a massive leukocytic infiltration, which varies depending on the disease duration and severity. In the initial phase which precedes the ulcer formation, monocytes and lymphocytes (mainly of the T type) together with single mast and plasmatic cells accumulate under the basal cell layer. In more advanced stages, polynuclear leukocytes dominate in the center of the ulcer, while on the lesion border the abundant mononuclear cell infiltration can be observed (Mills et al. 1980; Poulter and Lehner 1989). According to Poulter and Lehner (1989), this characteristic of the inflammatory infiltration is not exclusively specific to RAS and does not differ from the one observed in Behçet's syndrome, erythema multiforme, lupus erythematosus and traumatic ulcers.

**Fig. 2:**
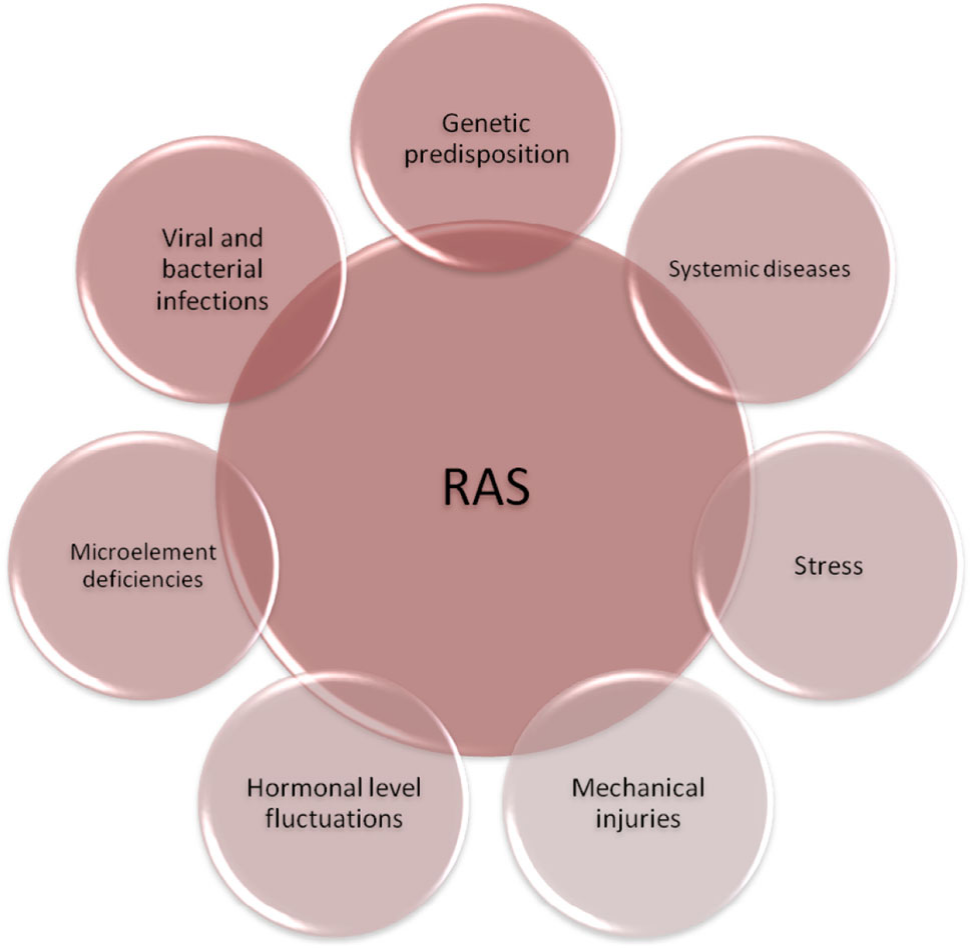
Modifying factors of the immunologic response in RAS

Factors that modify the course of the immune response in RAS are presented in Fig. 2.

## Genetic Predisposition

First reports on the role of genetic predisposition in the development of RAS are dated to the middle 1960s of the twentieth century. Based on the observation of the families suffering from RAS, Ship (1965) and Miller et al. (1977) propounded the autosomal recessive or multigene mode of inheritance with modifying influence of the environment. The role of genetic factors in the etiopathogenesis of recurrent aphthae was confirmed in further studies of relatives and twins with RAS, where the positive family history of the disease was reported in 24–46 % of cases (Rogers 1997; Scully and Porter 2008). The disease in parents significantly influences the risk of RAS and the course of the condition in their children—patients with a positive family history of RAS suffer more frequent recurrences and more severe course of the disease comparing to those with a negative RAS family history (Almoznino et al. 2013; Lehner 1978; Yilmaz and Cimen 2010). Moreover, in both, RAS and Behçet's syndrome, the risk of the disease development was higher in monozygotic twins than in dizygotic ones (Kobayashi et al. 2005; Yilmaz and Cimen 2010). The genetic risk factors that modify the individual susceptibility to RAS include various DNA polymorphisms distributed in the human genome, especially those related with the alterations in the metabolism of interleukins (IL-1β, IL-2, IL-4, IL-5, IL-6, IL-10, IL-12), interferon (IFN)-γ and tumor necrosis factor (TNF)-α (Akman et al. 2006, 2008; Bazrafshani et al. 2002, 2003; Buño et al. 1998; Guimarães et al. 2006, 2007; Hall et al. 2000; Kalkan et al. 2013; Pekiner et al. 2012). The role of DNA polymorphisms in serotonin transporter gene, endothelial nitric oxide synthase gene and cell adhesion molecule genes has also been considered (Alkhateeb et al. 2013; Gallo et al. 2012; Karasneh et al. 2005, 2009; Kim et al. 2003; Oksel et al. 2006; Salvarani et al. 2002; Victoria et al. 2005; Wang and Wang 2000). Other researchers reported the correlation between the selected HLA allele and the increased risk of RAS and Behçet’s syndrome (Albanidou-Farmaki et al. 2008; Challacombe et al. 1977; Sun et al. 2001). In patients with RAS, a higher incidence of HLA-A33, HLA-B35 and HLA-B81 (Wilhelmsen et al. 2009), HLA-B12 (Lehner et al. 1982), HLA-B51 (Özdemir et al. 2009), HLA-DR7 and HLA-DR5 and lower incidence of HLA-B5 and HLA-DR4 (Albanidou-Farmaki et al. 1988; Gallina et al. 1985) was observed when compared to healthy controls.

## Bacterial and Viral Infections

Among the potential factors with the ability to modify the immunologic response and to invoke recurrent aphthae in predisposed subjects some authors mention bacterial (*Streptococcus oralis*, *Helicobacter pylori*) and viral (herpes simplex virus, varicella-zoster virus, cytomegalovirus, adenoviruses) antigens. The studies’ results, however, are ambiguous and conflicting (Barile et al. 1963; Donatsky 1976; Natah et al. 2004; Shimoyama et al. 2000; Sook-Bin and Sonis 1996). The elevated serum level of antibodies against some streptococcal strains in RAS patients reported in the 1960s was not confirmed in latter studies, similarly in the case of antibodies against *H. pylori* (Barile et al. 1963; Donatsky 1976; Fritscher et al. 2004; Mansour-Ghanaei et al. 2005; Shimoyama et al. 2000). Tas et al. (2013) proved the beneficial effect of *H. pylori* eradication in patients with RAS. The underlying mechanism, however, is rather related with the increase in serum vitamin B12 levels after the eradication than with the direct action of the bacteria. The attempts to isolate herpes simplex, cytomegalovirus, varicella-zoster and Epstein-Barr viral DNA from the biologic material collected from aphthae and mononuclear peripheral blood cells were successful only in single case of RAS, which also does not confirm the direct role of viruses in the etiopathogenesis of the condition (Natah et al. 2004). Also Greenspan et al. (1985) concluded that neither cell-mediated hypersensitivity to streptococcal or viral antigens nor cross-reactivity between oral mucosal and streptococcal antigens is likely to play a role in the pathogenesis of RAS.

## Food Allergies and Microelement Deficiencies

In some patients with RAS, the deficiency in hematins (iron, folic acid, vitamin B12) was revealed (Khan et al. 2013; Lopez-Jornet et al. 2013; Natah et al. 2004; Olson et al. 1982; Scully and Porter 2008; Sook-Bin and Sonis 1996; Volkov et al. 2009); however, their modifying influence on the course of the immune response in RAS seems to be limited. In research by Lalla et al. (2012), Nolan et al. (1991a, b), Porter et al. (1992) and Haisraeli-Shalish et al. (1996), the supplementation of lacking microelements modified the disease course only in a small percentage of patients. Contrary, Volkov et al. (2009) observed positive effects of the oral vitamin B12 supplementation in RAS subjects regardless of the initial serum levels of this microelement. Some reports on the role of zinc deficiency in RAS were also published. Up to now, the theory was not unequivocally confirmed and the studies’ results are conflicting (Endre 1991; Pang 1992).

According to some researchers, also the exposition to some food ingredients, e.g., chocolate, gluten, cow milk, preservatives, nuts and food coloring agents may induce the pro-inflammatory cascade in RAS (Natah et al. 2004; Eversole et al. 1982; Sook-Bin and Sonis 1996; Wardhana 2010). In some patients, the clinical improvement was observed after inducing the elimination diet. In their double-blind study, Hunter et al. (1993) concluded that also the placebo-effect probably modifies the course of RAS—the clinical improvement was observed in both study groups: patients on real elimination diet and patients on a regular diet, exposed to potential trigger food ingredients. Meanwhile, Tarakji et al. (2012) did not confirm any important role of dietary habits in development of RAS.

## Systemic Diseases and Hormonal Imbalance

Based on many studies’ results, recurrent aphthae appear more frequently in patients with gastro-intestinal disturbances, mainly those from the group of chronic inflammatory bowel diseases (Crohn’s disease, ulcerative colitis) and celiac disease (Aydemir et al. 2004; Hunter et al. 1993; Olszewska et al. 2006; Rogers 1997; Scully and Porter 2008). This correlation may partially result from the food and microelement deficiencies—a characteristic complication in this group of the diseases (Natah et al. 2004). The coincidence of aphthae with inflammatory bowel diseases and celiac disease may also be related with autoimmune reactions, assumed as a background of all the mentioned conditions (Woźniak-Stolarska et al. 2003). Aphthae were also a frequent finding in HIV-infected persons, who manifested the disproportion of CD4 and CD8 lymphocytes together with decreased neutrophil count (MacPhail et al. 1991; Miziara et al. 2005; Muzyka and Glick 1994; Nesti et al. 2012).

Some reports mention also the correlation between the serum levels of sexual hormones and the course of RAS. Exacerbations of the condition were observed mainly in the luteal phase of the menstrual cycle and during the menopause, while the remissions seem to appear often during pregnancy and in women on contraceptives (Natah et al. 2004; Scully et al. 2003; Sook-Bin and Sonis 1996).

## Mechanical Injuries

In many RAS-predisposed patients, the lesions appear on the oral mucosa shortly after mechanical irritation of the area; however, the mechanism of this reaction remains not fully understood (Natah et al. 2004, Scully et al. 2003, Sook-Bin and Sonis 1996, Wray et al. 1981). Polańska et al. (2006) suggested the role of neutrophil elastase in the process of post-traumatic formation of aphthous ulcer. On the other hand, based on the epidemiologic observations, most of the researchers indicate the lower incidence of RAS in smokers in comparison to non-smoking subjects, with a correlation with habit duration and severity (Axell and Henricsson 1985; Bittoun 1991; Sawair 2010; Tuzun et al. 2000). That could be explained by a higher level of oral mucosa keratinization in response to smoking, which makes it less prone to injury and irritation (Natah et al. 2004; Scully et al. 2003; Sook-Bin and Sonis 1996). Nicotine and its metabolites can also decrease the level of pro-inflammatory cytokines (TNF-α, IL-1 and IL-6) and increase the level of anti-inflammatory IL-10 (Subramanyam 2011). Also smokeless tobacco products seem to decrease the risk of RAS development (Grady et al. 1992).

## Stress

Another described factor potentially related with RAS exacerbations is stress (Natah et al. 2004; Keenan and Spivakovksy 2013; Scully et al. 2003; Sook-Bin and Sonis 1996; Volkov et al. 2009; Zadik et al. 2012). According to some authors, it rather triggers the onset of the episode than influences its duration (Huling et al. 2012; Keenan and Spivakovksy 2013). Psychogenic effects modify the immune response also in the other conditions with a suspected autoimmune background, like lichen planus and chronic inflammatory bowel diseases (Soto Araya et al. 2004).

## Mechanisms of the Immune Response in RAS

**Fig. 3:**
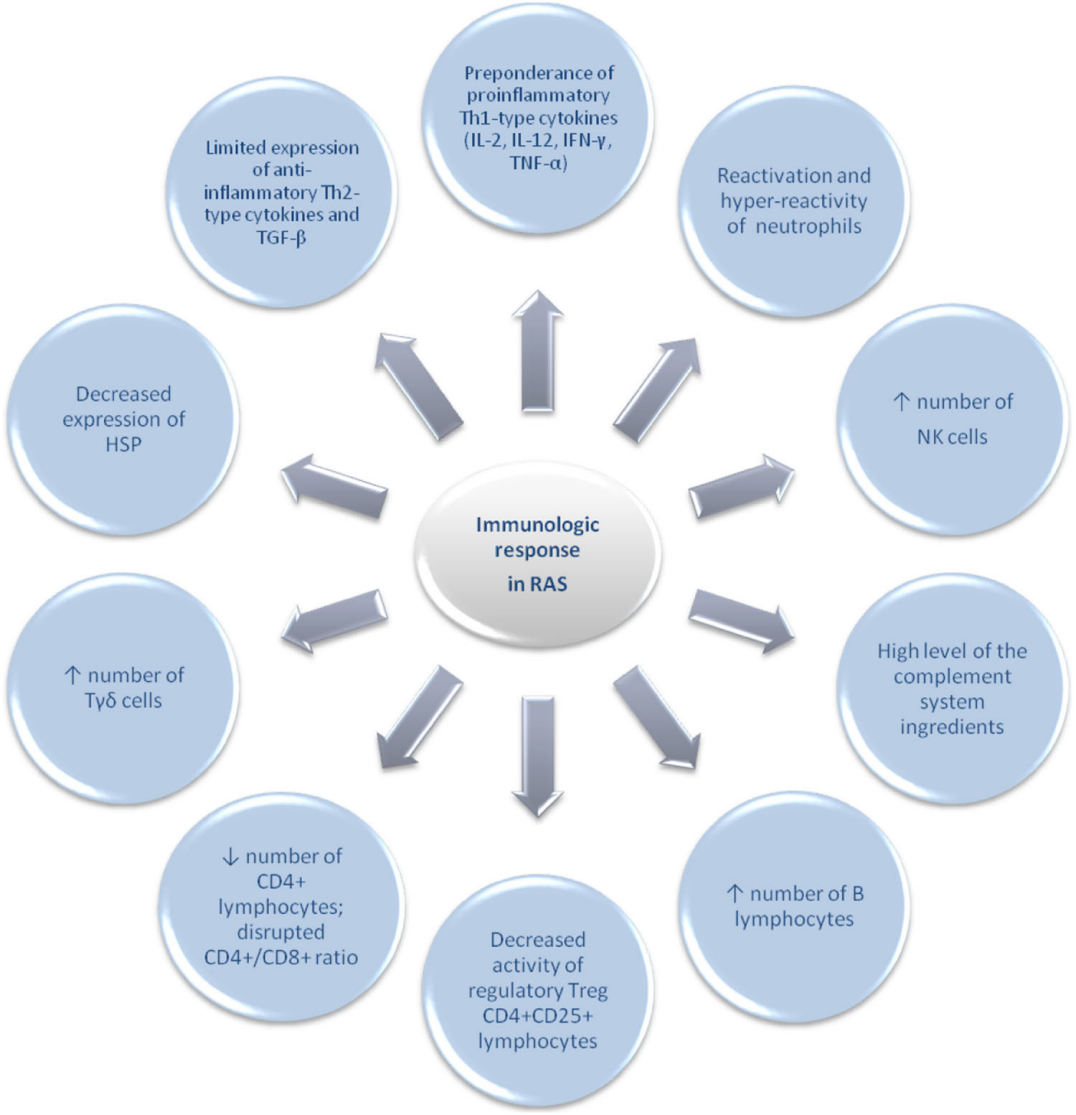
Mechanisms of the disrupted immunologic response in RAS

The basic mechanisms of the immunologic response disruption in RAS are illustrated in Fig. 3.

One of the important factors that may induce and determine the type of the immune response in the human body is the cytokines (Konopka et al. 1995). Th1 type cytokines, which include: IL-2, IL-12, IFN-γ and TNF-α, determine the predisposition towards autoimmunisation, induce the cellular type response and stimulate the secretion of IgG. Th2 type cytokines, including: IL-4, IL-5, IL-10 and IL-13, manifest anti-inflammatory properties, stimulate the humoral immune response and the secretion of IgE. Strong anti-inflammatory effect is contributed also to another cytokine called transforming growth factor (TGF)-β, secreted mainly by the T-regulator lymphocytes (Buño et al. 1998; Lewkowicz et al. 2004). It was found that aphthous ulcer develops in response to the enhanced immunologic reaction against particular regions of the oral mucosa (Borra et al. 2004; Eversole 1994; Lewkowicz et al. 2004). This reaction occurs in a result of improperly initiated cascade of cytokines, which activate certain immune processes (Borra et al. 2004; Lewkowicz et al. 2004). It was found that in patients with RAS, the immune system’s function become disrupted in response to some kind of not yet defined trigger factor, which may include viral and bacterial antigens or stress. In many reports also the role of the autoimmunisation in the disease development was emphasized (Borra et al. 2004; Górska 1997b; Hietanen et al. 2012; Malmström et al. 1983). Both types of the immune response: natural and acquired (humoral and cellular) may become disturbed in patients with RAS, which, among the others, manifests with neutrophils’ reactivation and hyper-reactivity, elevated concentration of the complement ingredients, increased number of NK cells and B lymphocytes, disrupted CD4^+^/CD8^+^ ratio and increased number of CD25^+^ and T cell receptor (TCR) γδ cells in peripheral blood (Eversole 1997; Lewkowicz et al. 2003; Nowak and Górska 2008). Therefore, to determine the mechanism of the immunologic response in RAS, it was necessary to define the cytokine profile in patients suffering from this disease. Many authors suggest that the Th1 type immunologic response is the one to play the crucial role in the development of RAS (Albanidou-Farmaki et al. 2007; Borra et al. 2004; Buño et al. 1998; Lewkowicz et al. 2004, 2011).

Significantly higher secretion of Th1 cytokines in RAS patients in comparison to the controls was described by Lewkowicz et al. (2004). The increased production of IL-2, IFN-γ and TNF-α by the peripheral blood mononuclear cells was observed both in the acute phase of the disease and in the remission. Meanwhile, the secretion of anti-inflammatory cytokines TGF-β and IL-10 was significantly decreased in RAS patients compared to the healthy controls. This observation confirms that the imbalance in pro- and anti-inflammatory cytokines’ production may contribute to the development of autoimmunisation and RAS in predisposed subjects. Similarly, Albanidou-Farmaki et al. (2007) found the increased number of T lymphocytes able to produce pro-inflammatory cytokines (IL-2, IL-12 and IFN-γ) and the decreased number of IL-10-producing cells in peripheral blood of the RAS patients in comparison to the healthy controls. Also Borra et al. (2004) demonstrated the increased expression of Th1 type response gene cluster in comparison to Th2 gene cluster in patients with RAS. The authors emphasized that also some physiologic states or therapeutics (e. g. pregnancy, nicotine exhibition, glucocorticoid, thalidomide and tetracycline treatment) are well known as the Th1 type immunologic response inhibitors; therefore, they may influence the course of RAS. Increased activity of the Th1 type immunologic response was also observed in some other autoimmune-mediated conditions like Crohn’s disease, celiac disease and PFAPA (periodic fever, aphthous stomatitis, pharyngitis and adenitis) syndrome (Yao and Furst 2008). Apart from the decreased expression of anti-inflammatory cytokines, significantly decreased expression of heat shock protein 27 was observed in the oral mucosa of the RAS patients (Miyamoto et al. 2008). Heat shock proteins manifest the property to inhibit the expression of pro-inflammatory cytokines and they participate in the inhibition of the monocytes differentiation into the dendritic cells. The decrement in the concentration of that protein fraction may, therefore, exacerbate the inflammation. The elevated levels of heat shock protein were observed in tobacco smokers, which may partially explain the phenomenon of the lower incidence of aphthae in smokers compared to non-smokers (Miyamoto et al. 2008). Moreover, in the latter study, Lewkowicz et al. (2008) demonstrated the decreased efficacy of Treg CD4^+^CD25^+^ cells in the inhibition of the pro-inflammatory cytokines’ secretion by CD4^+^ effector T lymphocytes in the RAS patients compared to the controls with no systemic diseases.

Buño et al. (1998) attempted to determine the concentration of pro-inflammatory cytokines directly in the oral mucosa of patients with RAS and they demonstrated significantly increased levels of IL-2, IL-4, IL-5, IFN-γ and TNF-α together with a decreased concentration of anti-inflammatory cytokine IL-10 in the examined group when compared to healthy controls. These results indicate that the complex mode of the immune response participates in the development of RAS. On the one hand, the authors demonstrated the elevated concentration of pro-inflammatory cytokines in the oral mucosa affected with aphthae (Th1 type response), but on the other hand the increased levels of Th2 type cytokines (IL-4, IL-5) were also detected.

Nowak and Górska (2008) compared the concentration of IL-2 in the peripheral blood and in stimulated saliva in patients with RAS and in healthy volunteers. They have demonstrated significantly increased levels of this pro-inflammatory cytokine in the blood samples of RAS patients in comparison to controls; however, no significant differences were detected in stimulated saliva. According to the authors these results suggests that IL-2 can be produced by T lymphocytes.

The role of TNF-α, another pro-inflammatory cytokine, in the development of aphthae was confirmed in the study by Natah et al. (2000a), who compared the number of TNF-α-containing cells in the oral mucosa affected by aphthae and in the mechanically injured oral mucosa bioptates. Cells rich in TNF-α were observed much more frequently in biologic material received from the RAS patients, which suggests the role of the tested cytokine in the lymphocytes activation and recruitment in the course of the disease. In the other study, Natah et al. (2000b) also found a significant elevation of TCR γδ cells number in the oral mucosa with aphthous lesions. Lymphocytes with T γ/δ cell receptors produce IL-2, manifest cytotoxic properties and destroy some certain virus-infected cells. They also play role in the process of epithelial growth control. Previous observations confirmed the elevated concentration of that type cells in the subjects with rheumatoid arthritis, tuberculosis and celiac disease. The results of Natah et al. (2000b) study revealed that the local elevation in TCR γδ cells number was also found in the oral mucosa in RAS patients. However, the biological role of those cells in the process of the aphthous ulcer formation and healing still remains not clearly understood. Similar conclusions were presented by Freysdottir et al. (1999), who observed the increased TCR γδ cells’ concentration in the peripheral blood of patients with Behçet’s syndrome and RAS.

In Table 3, we summarized the role of inflammatory mediators in the etiopathogenesis of RAS based on Polish and foreign studies.

**Table 3:** The role of inflammatory mediators in the etiopathogenesis of RAS and the mechanisms of the immune system disruption based on the literature reports

Mediator	Tissue studied	Mechanism of the immune system disruption in RAS	References
IL-2, IFN-γ, TNF-α	PBMC	↑ production	Lewkowicz et al. (2004); Poland
TGF-β, IL-10		↓ production	
IL-2, IL-12, IFN-γ	PBMC	↑ production	Albanidou-Farmaki et al. (2007); Greece
IL-10		↓ production	
IL-2, IL-4, IL-5, IFN-γ, TNF-α	Oral mucosa	↑ concentration	Buño et al. (1998); USA
IL-10		↓ concentration	
IL-2	Blood	↑ concentration	Nowak and Górska (2008); Poland
	Saliva	No differences between RAS subjects and healthy controls	
TNF-α	Oral mucosa	↑ concentration of TNF-α-containing cells	Natah et al. (2000a); Finland
	Oral mucosa	↑ concentration of TCR γδ lymphocytes	Natah et al. (2000b); Finland
	Blood	↑ concentration of TCR γδ lymphocytes	Freysdottir et al. (1999); Great Britain
	Oral mucosa	↑ expression of Th1 type response gene cluster compared to Th2	Borra et al. (2004); Brazil
IL-10	Oral mucosa	↓ tissue expression	Miyamoto et al. (2008); Brazil
HSP27			
		↓ Treg CD4^+^CD25^+^ efficacy in the inhibition of proinflammatory cytokines secretion by the effector CD4^+^ T lymphocytes	Lewkowicz et al. (2008); Poland

*PBMC* peripheral blood mononuclear cells, *HSP27* heat shock protein 27

The common occurrence of aphthae was also described in patients with HIV, who manifested the disturbed proportion of CD4 and CD8 lymphocytes (Aydemir et al. 2004). The decreased CD4:CD8 ratio has been also found in RAS patients not affected by HIV; studies results, however, are not consistent in this matter (Aydemir et al. 2004; Lewkowicz et al. 2008).

Ambiguous conclusions on the immunologic processes in RAS were also stated in studies performed with the use of immunofluorescent methods. Wilhelmsen et al. (2008) did not reveal the deposits of immune complexes in the oral mucosa specimens received from patients with RAS. Such deposits are characteristic for certain autoimmune-mediated vesiculo-bullous diseases, like *Pemphigus vulgaris* and *Pemphigoid*. The lack of immune complexes in RAS could, therefore, become an important differentiating marker between RAS and vesiculo-bullous diseases. Some other authors, however, observed the deposits of IgG, IgM and IgA in the epithelial *stratum spinosum* in RAS and in Behçet syndrome (Williams and Lehner 1977).

## Conclusion

The presented studies’ results confirm the crucial role of the immunologic disturbances in the etiopathogenesis of RAS. Conclusions from the genetic research support the thesis that the immune system’s hyper-reactivity in response to some trigger factors in patients with RAS is at least partially related with a genetic predisposition—certain polymorphisms in the cytokine encoding genes implicate a higher predisposition to RAS. As the etiopathogenesis of the condition has not been clearly defined, the treatment is mainly symptomatic and not very effective. Discovering the direct etiopathogenetic factors in RAS may in future help to predict the risk of the disease occurrence and to develop the effective, causative management. Inconsistent results of the multiple international studies focused on genetic and immunologic background of the condition and insufficient number of that type research on Polish population indicate a necessity to perform such an analysis also in our country.
